# Patient-derived tumor models are attractive tools to repurpose drugs for ovarian cancer treatment: pre-clinical updates

**DOI:** 10.18632/oncotarget.28220

**Published:** 2022-03-24

**Authors:** Magdalena Cybula, Magdalena Bieniasz

**Affiliations:** ^1^Oklahoma Medical Research Foundation, Aging and Metabolism Research Program, Oklahoma City, OK 73104, USA

**Keywords:** PDX, ovarian cancer, repurposed drugs, tumor models

## Abstract

Despite advances in understanding of ovarian cancer biology, the progress in translation of research findings into new therapies is still slow. It is associated in part with limitations of commonly used cancer models such as cell lines and genetically engineered mouse models that lack proper representation of diversity and complexity of actual human tumors. In addition, the development of *de novo* anticancer drugs is a lengthy and expensive process. A promising alternative to new drug development is repurposing existing FDA-approved drugs without primary oncological purpose. These approved agents have known pharmacokinetics, pharmacodynamics, and toxicology and could be approved as anticancer drugs quicker and at lower cost. To successfully translate repurposed drugs to clinical application, an intermediate step of pre-clinical animal studies is required. To address challenges associated with reliability of tumor models for pre-clinical studies, there has been an increase in development of patient-derived xenografts (PDXs), which retain key characteristics of the original patient’s tumor, including histologic, biologic, and genetic features. The expansion and utilization of clinically and molecularly annotated PDX models derived from different ovarian cancer subtypes could substantially aid development of new therapies or rapid approval of repurposed drugs to improve treatment options for ovarian cancer patients.

## INTRODUCTION

Ovarian cancer constitutes a diverse group of malignancies that are a leading cause of gynecological cancer deaths worldwide and the 5th most common type of cancer in women [1, 2]. Though early stage ovarian cancer is often curable, most patients are diagnosed at an advanced stage of the disease, where the tumor has already metastasized within pelvis and abdominal cavity [3, 4]. Despite advances in surgical and chemotherapeutic approaches, metastatic ovarian cancer is associated with swift disease progression and death, which poses a significant burden on public health and the cost of health care [3–7]. There is an urgent need to improve ovarian cancer therapy, but the development of anticancer drugs is a lengthy and expensive process. Repurposing existing approved drugs without primary oncological purpose is a promising alternative to new drug development to address unmet need in ovarian cancer [5, 8]. Studies evaluating novel pharmacological effects of existing drugs could permit more rapid identification of new therapies [9, 10]. These approved agents including antibiotic, antiparasitic, antiinflammatory, or antidiabetic agents have known pharmacokinetics, pharmacodynamics, and toxicology. Thus, if effective, repurposed drugs could be approved as anticancer agents at lower cost, in comparison to new drugs [11]. The successful translation of repurposed drugs to clinical application requires an intermediate step of pre-clinical animal studies to validate efficacy of drugs *in vivo* [12]. The mouse is the most common animal model for pre-clinical studies, due to the similarity of the mouse and human genomes [13]. However, even with promising results in animal models, only ~5% of potential anticancer agents demonstrate sufficient clinical efficacy in phase III trials to receive approval for clinical use [14]. This challenge is associated in part with the lack of pre-clinical cancer models that faithfully replicate human tumor diversity, heterogeneity, and patient’s response to treatment [15]. Development of new therapies will benefit from improved pre-clinical models capable of predicting patient subsets that will respond to a particular anticancer agent.

In recent years, patient-derived xenograft (PDX) models have emerged as a powerful tool in oncology for the development of novel therapies for early, advanced, and drug-resistant tumors, as well as the implementation of personalized cancer therapies [16–20]. PDXs are renewable cancer models engrafted in mice, developed from fresh human tumors without prior *in vitro* manipulation. These models are one of the most clinically relevant tumor models, as they retain key characteristics of the original patient’s tumor, including histologic, biologic, and genetic features. Tumor-specific characteristics are maintained through multiple mouse-to-mouse passages [16, 18]. In addition, PDXs recapitulate the dynamics of tumor evolution and patient’s response to therapy with high fidelity [18, 21, 22]. PDX models have been used to evaluate new therapeutic approaches and to perform pre-clinical drug testing and biomarker identification [23]. Recently, several reports have described utilization of PDX models of ovarian cancer to study antitumor potential of repurposed drugs [24–26]. However, *in vivo* responses to standard of care chemotherapy and/or repurposed therapeutics have been reported almost solely for a common ovarian cancer subtype high-grade serous ovarian cancer (HGSOC) [27, 28]. Rare ovarian cancer subtypes pose challenges to research due to their low incidence, and only limited attempts have been made to develop PDX models from those malignancies [29].

In this review, we describe recent advances in the establishment of PDX models derived from a variety of ovarian cancer subtypes and discuss their application for pre-clinical evaluation of repurposed drugs.

## OVARIAN CANCER

Ovarian cancer is a deadly disease affecting 11.3 per 100,000 women per year in the US [30]. In 2021, there will be approximately 21,410 new diagnoses of ovarian cancer, and 13,770 American women will die from this devastating disease [31]. The prognosis for patients depends on the stage of the cancer at the time of diagnosis; the 5-year survival rate for stage I disease is 80%, but drops to only 20% for stage IV [5]. Ovarian cancer development risk rises with age; about 50% of women is 63 years old or older at the diagnosis.

**Figure 1:**
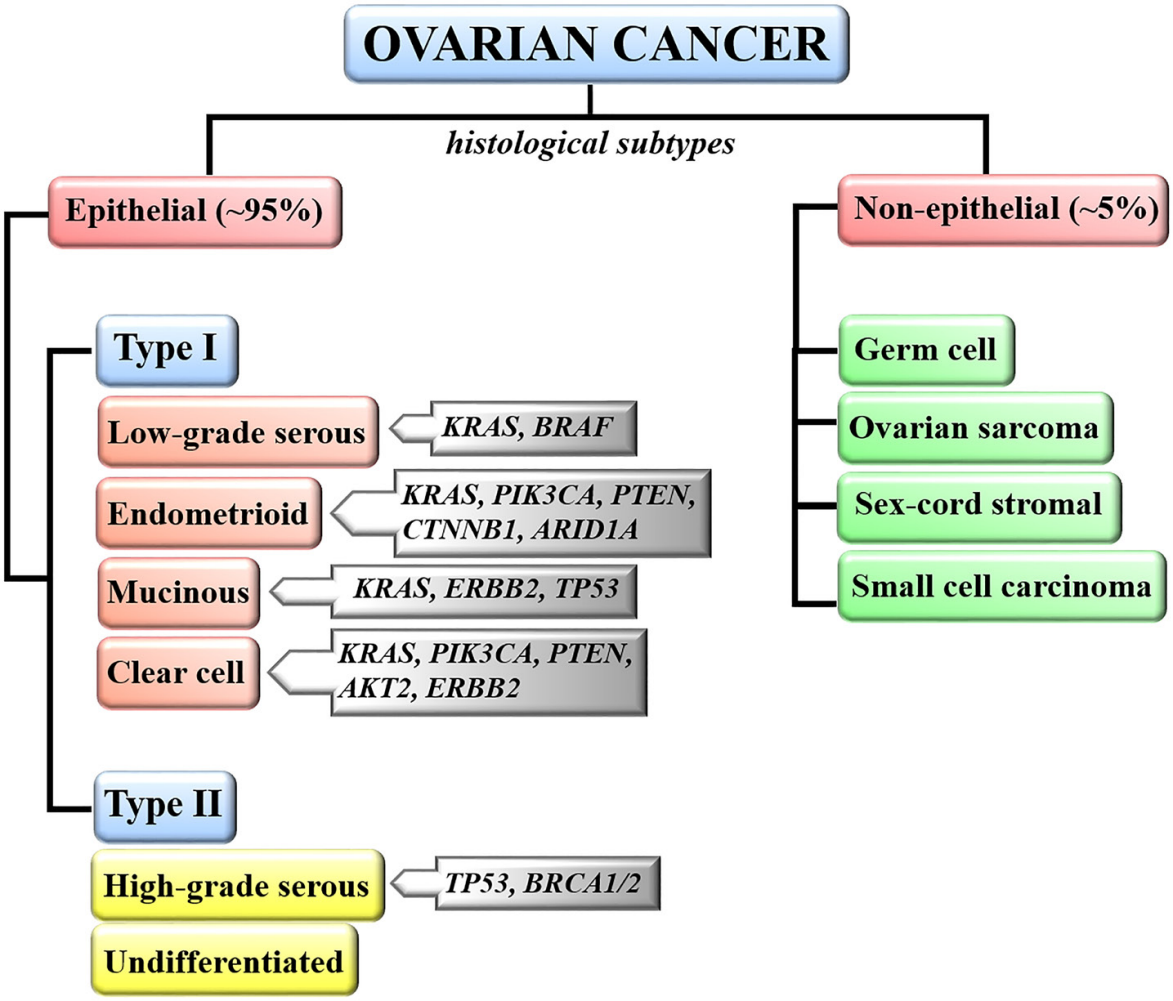
Classification of ovarian malignancies. Ovarian neoplasms are classified based on tumor histology, grade and molecular evidence that type I and type II tumors develop through different pathways. Most frequently altered genes in each ovarian cancer subtype are indicated.

Ovarian cancer is categorized into two main groups including epithelial ovarian cancers (EOCs) and non-epithelial tumors representing 95% and 5% of all ovarian malignancies, respectively [5] (Figure 1). Non-epithelial tumors include germ cell, sex-cord stromal cancers, small cell carcinoma, and ovarian sarcoma. EOC is a heterogeneous group of cancers, including neoplasms that differ in molecular, histological, and clinicopathological features. Histologically, there are four EOC subtypes including serous, mucinous, endometrioid, and clear cell ovarian cancer. Serous ovarian cancer is the most common subtype and is further classified as Type I – low-grade serous ovarian cancer (LGSOC, 5–10% of EOC), and Type II – high-grade serous ovarian cancer (HGSOC, 70–90% of EOC) [1]. Mucinous, endometrioid and clear cell ovarian cancers represent 3%, 10%, and 10% of epithelial ovarian carcinomas, respectively (Figure 1) [30].

### High-grade serous ovarian cancer (HGSOC)

HGSOC is the most frequent histotype of ovarian cancer. The cell of origin of this tumor subtype has been extensively debated. Initially, HGSOC was thought to arise from ovarian surface epithelium (OSE), however, in the last decade, several comprehensive studies provided evidence that the majority of HGSOCs originate from the distal fallopian tube epithelium (FTE) [1, 32, 33]. It has been shown that STIC (serous tubal intraepithelial carcinoma) lesions developing in fallopian tubes are premalignant precursors of HGSOCs. Most STICs exhibit robust immunostaining of p53 and harbor *TP53* mutations (defined as “p53 signature”) [34, 35]. In addition to the histopathological resemblance, STIC lesions exhibit genomic instability and identical *TP53* mutations as their corresponding invasive HGSOCs [1, 36]. Furthermore, genomic analysis of STICs, fallopian tube tumors, ovarian tumors, and peritoneal metastases from the same patients confirmed an evolutionary relationship, suggesting that STIC lesions are likely early events in the progression of HGSOC [37]. The progression of STIC into malignant HGSOC is associated with dissemination of cancer cells from the fallopian tube to the ovary. The cancer cells that colonize the ovary have already acquired the genetic mutations of *TP53, BRCA1, BRCA2,* and PI3K pathway genes that are key drivers of HGSOC progression [1]. Furthermore, it has been shown that in women carrying *BRCA* mutations who underwent prophylactic bilateral salpingo-oophorectomy (removal of FTs and ovaries), the procedure reduced the risk of ovarian cancer development to less than 5% [38]. Similarly, different studies reported that prophylactic or opportunistic bilateral salpingectomy (removal of fallopian tissue) is associated with 29.2% to 64% decrease in risk of developing ovarian cancer [38, 39]. Nevertheless, some studies support the dual origin of HGSOC, where FTE is a tissue of origin of the majority of these tumors, while a small proportion of HGSOCs arises from OSE. For instance, it has been shown that some subgroups of HGSOC, in particular *BRCA* wild-type tumors are molecularly more similar to OSE [40]. Hao et al. performed gene expression profiling of a large collection of FTE, OSE and HGSOC tissues and demonstrated that the tumor molecular signature indicates the co-existence of ovary-derived and FTE-derived tumors, which accounts for about 40% and 60% of all HGSOCs, respectively [41]. In different study, Ducie et al. reported that up to 12% of HGSOCs show greater transcriptional similarity to OSE than to FTE [33]; similar results were also reported by others [42]. In addition, proteomic studies also support the dual origin of HGSOC and suggest that OSE-derived tumors are more aggressive and have worse prognosis [43]. In summary, based on current knowledge, FTE has been considered as a predominant tissue of origin of HGSOC, however, the existence of HGSOC subpopulations with OSE-like molecular signatures or no evidence of fallopian tube involvement in tumor development raises the possibility that HGSOCs could originate from more than one tissue.

Mutations of *TP53* are a nearly universal characteristic of HGSOC, being reported in about 96% of cases [44]. Furthermore, this ovarian tumor subtype demonstrates an extraordinary degree of genomic alterations associated with amplification or loss of a large number of genes. High genome instability can be explained by the fact that this neoplasm frequently displays mutations, as well as promotor methylations in DNA repair genes, including homologous recombination components like *BRCA1* and *BRCA2* [2, 44]. Such alterations are known to cause homologous repair deficiency (HRD), which is reported in about 50% of HGSOC [44]. HGSOC affects older women (average age at diagnosis is 63 years), and it is usually diagnosed in advanced stage, which is associated with poor overall prognosis [2].

### Low-grade serous ovarian cancer (LGSOC)

LGSOC was recently distinguished from HGSOC as a separate subtype of serous ovarian malignancy [45, 46]. LGSOC shows distinct genetics and clinicopathological features and arises from a benign serous neoplasm cystadenoma/adenofibroma, progress to serous ovarian borderline tumor (SBOT), then to non-invasive LGSOC, and finally to LGSOC [46]. The most common aberrations found in LGSOC are mutations of *KRAS* (15.4–54.5%) and *BRAF* (0–32%) genes [47]. *TP53* mutations are rare in LGSOC [48], as are *BRCA* mutations [46]. This type of malignancy is associated with low-level copy number alteration, in contrast to HGSOC [49]. It was also reported that the MAPK (mitogen-activated protein kinase) pathway plays a crucial role in the pathogenesis of LGSOC [50]. This subtype of EOC is typically diagnosed at a younger age (43–55 years) and associated with longer overall survival when compared with HGSOC [47].

### Mucinous ovarian cancer (MOC)

MOC is distinct from other types of ovarian tumors, as it occurs in younger patients and 65–80% of cases are diagnosed at an early stage of the disease [51–53]. The etiology of MOC remains unclear, since normal mucinous epithelium does not exist within the ovary. MOC progression is a stepwise process, from benign mucinous cystadenoma, through borderline mucinous tumors, to mucinous carcinoma [52, 53]. The most frequently observed molecular alterations in MOC are *KRAS* mutations (40–60% of cases). *TP53* mutations and *ERBB2* amplification are also frequently reported, in 50% and 18% of tumors, respectively [54–57]. In contrast to HGSOC subtype, MOC lacks genomic alterations in the *BRCA* genes [53].

### Endometrioid ovarian cancer (ENDOC)

ENDOC is usually diagnosed at an earlier stage and younger age, resulting in a good prognosis [58, 59]. ENDOC arises from endometriosis, benign neoplasm or endometrioid borderline tumors. This type of malignancy frequently occurs with endometrioid endometrial cancer (EEC) [58, 60, 61]. It has been reported that ENDOC and EEC have similar molecular alterations and are clonally related [62]. ENDOC with synchronous EEC consist of EEC cells disseminated to the ovary [58, 62, 63]. The analysis of the molecular landscape showed that the mutational frequencies of *KRAS*, *PIK3CA*, *PTEN*, *CTNNB1*, and *ARID1A* are different in pure ENDOC, when compared to ENDOC with concomitant EEC. The *PIK3CA*, *PTEN*, *CTNNB1*, and *ARID1A* mutations are less prevalent, while *KRAS* is more frequently mutated in pure ENDOC in comparison to ENDOC with synchronous EEC. The differences in the molecular landscape and mutation frequency between pure ENDOC and ENDOC with synchronous EEC have to be taken into consideration when designing therapeutic strategies for the treatment of these malignancies [58].

### Clear cell ovarian cancer (CCOC)

Clear cell ovarian cancer is rare (up to 10% of all EOC cases), though a higher percentage of CCOC has been reported in East Asia [64, 65]. CCOC is typically diagnosed at a younger age and earlier stage, which is associated with good patients’ prognosis. However, when CCOC is diagnosed at an advanced stage, it has the worst prognosis of all epithelial ovarian carcinomas, due to its resistance to platinum-based chemotherapy. Like ENDOC, CCOC is associated with endometriosis [59, 66]. CCOC is reported to be associated with mutations of the *ARID1A* (up to 50%) and *PIK3CA* (33–50%) genes, loss of *PTEN* expression (40–51%), amplification of *AKT2* (14%), and amplification and overexpression of *ERBB2* (9.3–14%) [66–68]. Mutations of the *TP53* and *BRCA* genes are uncommon in this type of ovarian malignancy [64, 67].

## PATIENT-DERIVED XENOGRAFT OVERVIEW

**Figure 2:**
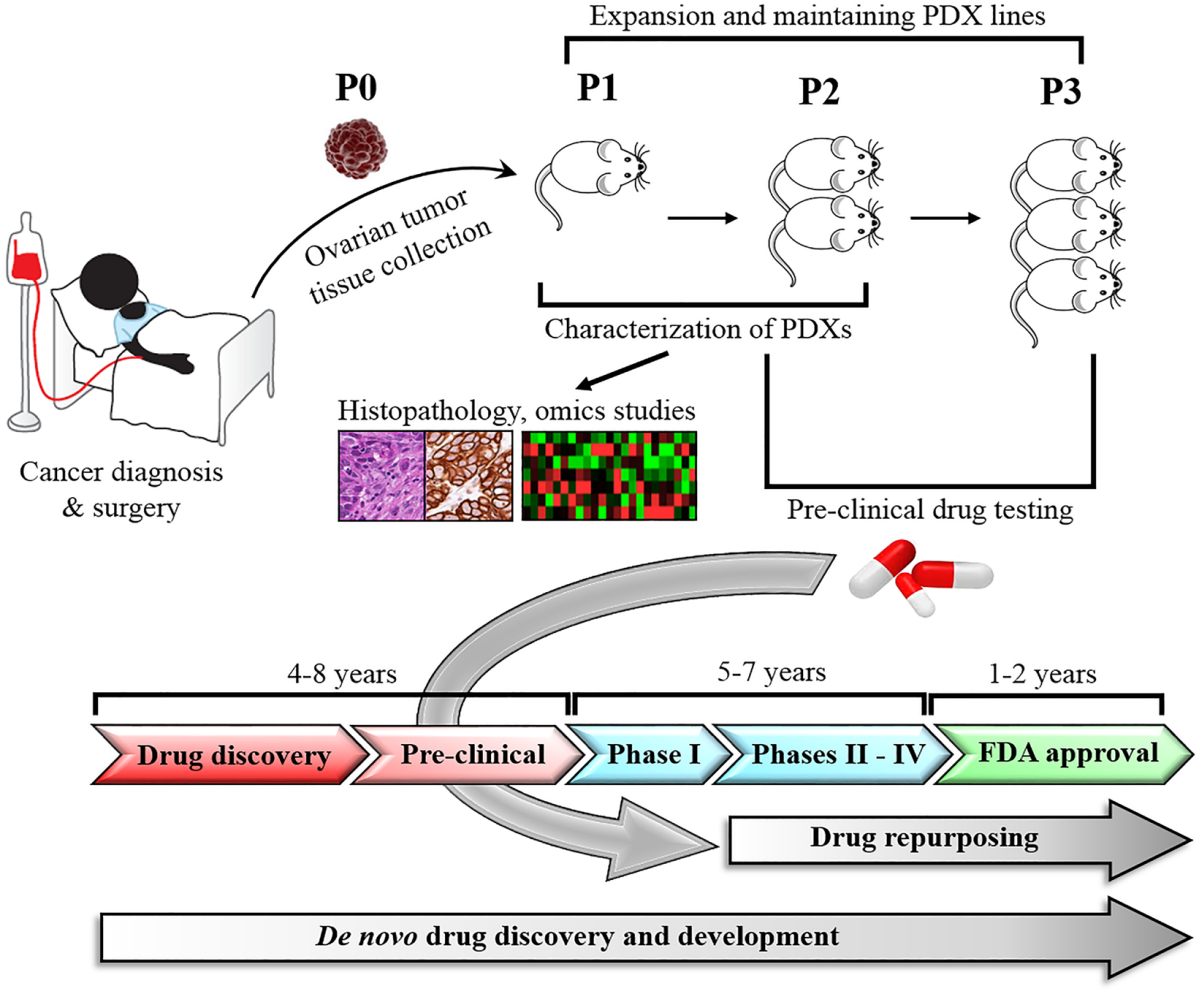
Development and application of PDX models in drug repurposing. Ovarian cancer tissues harvested from patients are engrafted directly into immunodeficient mice, expanded through serial passages, and histologically and molecularly characterized. Established PDX models are used in pre-clinical studies to test and validate new or repurposed drugs. In contrast to *de novo* anticancer drug development, which takes 10–17 years, drug repurposing takes only 6–9 years bypassing several steps that have been completed for the original drug indication.

A major obstacle in oncology is a scarcity of pre-clinical models that faithfully recapitulate the heterogeneity of human tumors, which poses a challenge to anticancer drug development and testing [15]. Although conventional models like cancer cell lines and cell line-based mouse xenografts are widely used tools for cancer research, they have poor predictive power for clinical response [16]. To overcome these barriers, significant efforts in development of more advanced tumor models, including patient-derived xenografts (PDXs) have been implemented in recent years [16, 18, 69]. PDXs preserve the heterogeneity of human tumors more accurately, and maintain cell-to-cell and cell-to-matrix interactions better compared to other cancer models [16, 64]. In addition, PDXs offer unique opportunities to study carcinogenesis, drug discovery and validation, since they are established by engrafting an intact patient tumor tissue in mice without previous *in vitro* culturing [70, 71]. Established PDX models can be propagated for multiple passages and serve as a valuable source of tumor tissue for multiple independent studies (Figure 2) [18, 72]. The first reported mouse xenograft model was established by subcutaneous implantation of human colon adenocarcinoma tissue into nude mice [69]. The same mouse strain and technique was utilized to develop the first ovarian cancer xenograft model in the late 1970’s [72]. Over the years, PDX model development has been improved by increasing the engraftment rate of human tumor tissue into mouse. The engraftment rate improvement was associated with the use of mouse strains with a higher level of immune suppression, as well as better techniques of tumor implantation.

The engraftment rate of epithelial ovarian cancer subtypes ranges from 25–95% [18, 73, 74]. The engraftment rate of a common HGSOC subtype is high (80–90%) as compared with other EOC subtypes (Table 1) [27, 73]. The establishment of HGSOC PDX models is relatively easy, since the success of tumor engraftment is associated with the high-grade histology and advanced disease stage [75]. There are a few reports about development of PDX models from rare EOC subtypes, usually established as part of a larger panel of EOC PDXs [75–77]. The median time of PDX development ranges from 1 to 12 months and depends on the ovarian cancer subtype and site of tumor implantation [76, 77]. NSG, SCID, and nude mice are the most commonly used immunodeficient mouse strains for development of EOC PDX models [27, 74, 76].

**Table 1:** The establishment rate of PDX models derived from different ovarian cancer subtypes

Tumor type	Mice strain	Implantation site	Engraftment rate (%)	No of PDXs	References
**High grade serous**	NSG NSG, NOD/SCID nude NSG NSG, NOD/SCID nude SCID/Beige nude nude	SQ, IB SQ, IB IP FT/ovary MFP SQ, IP, IB IP interscapular fat pad SRC	83 >90 31 >90 >90 ND 82 54 49	12 9 29 40 38 15 111 28 20	[27] [73] [80] [85] [79] [74] [75] [86] [77]
**Low grade serous**	SCID/Beige	IP	100	1	[29]
**Mucinous**	nude nude SCID/Beige nude	SQ SQ, IP, IB IP interscapular fat pad	100 ND 50 50	2 2 2 1	[117] [74] [75] [86]
**Endometrioid**	nude SCID/Beige	SQ IP	100 64	5 11	[74] [75]
**Clear cell**	nude nude SCID/Beige nude	SRC SQ, IP, IB IP interscapular fat pad	50 ND 90 100	2 2 10 1	[77] [74] [75] [86]

Abbreviations: IB: intrabursal; IP: intraperitoneal; FT: fallopian tube; MFP: mammary fat pad; SRC: subrenal capsule; SQ: subcutaneous; ND: not determined.

### PDX engraftment

The most common method to generate PDX model is to engraft a fragment of human primary tumor or metastatic tissue into immunodeficient mouse [16, 74, 78, 79]. Other specimens, like ascites can also be used to generate PDX models of ovarian cancer [80]. Heo et al. indicated several factors that affect ovarian tumor engraftment such as the quality and size of tumor tissue, as well as the abundance of tumor cells in the primary tumor sample [77]. Moreover, the process of human tumor sample collection and storage is also a crucial factor in PDX development, which can significantly affect tumor engraftment success. Reduction of the time between sample collection and implantation (ideally within 30–60 minutes) [17], as well as shortening the duration of surgical procedure of tumor implantation into mouse, is recommended. Tumor specimens that cannot be implanted immediately after excision should be properly stored by cryopreservation, though the engraftment rate of frozen-thawed specimens is lower than the engraftment of fresh tumor tissue. In addition, the type of cryoprotectant considerably affects the engraftment efficiency [81–83]. Alkema et al. tested two cryopreservation biobanking methods of patients’ ovarian tumor tissues and tumor tissues from established PDX. The authors tested high fetal calf serum (FCS)-based medium (95% FCS/5%DMSO) and low fetal calf serum (FCS)-based medium (10% FCS) that contained three different concentrations of DMSO, propanediol, polyvinylpyrrolidone, and ethylene glycol. The authors demonstrated that high FCS-based medium preserved tumor tissue better compared to low FCS-based medium (engraftment rate of primary tumors was 67% and PDXs 94% for high FCS-based medium, and 38% and 67%, for low FCS-based medium, respectively) [81]. Moreover, tumor samples originating from advanced stage cancers had higher tumor take rate, compared to samples from less aggressive malignancies [75]. The implantation of solid tumor fragments was more successful than the implantation of a suspension of single cancer cells after tumor dissociation, perhaps because it preserved tumor architecture, and successful engraftment could be achieved more quickly [17]. Tumor engraftment rate depends also on the implantation site [17]. Ovarian tumor tissue can be implanted into mice subcutaneously (heterotopic), intraperitoneal (IP) to recapitulate advanced disease, and engrafted to the fallopian tube fimbria/ovary (orthotopic). In the orthotopic method, the tumor sample is implanted into the same anatomical site as the patient tumor. PDXs established by the implantation of tumor fragments into the fallopian tube fimbria/ovary are more physiologically relevant models, and thus more likely to develop metastasis and ascites. Some authors successfully utilized intrabursal implantation (IB) to establish ovarian PDXs [27, 73, 74]. However, since HGSOC subtype predominantly develops from the fallopian tube rather than the ovary, and due to the lack of ovarian bursa in humans [84], it seems more appropriate to consider intrabursal xenograft as heterotropic model, at least in the context of modeling the primary tumor.

George et al. used NSG mice to establish an orthotopic PDX collection and reported a 93% take rate of HGSOC tissues [85]. The inoculation and tumor growth monitoring of orthotopic ovarian xenografts is technically more challenging than the subcutaneous models, thus the latter is often preferred for its simplicity [17, 78]. The implantation of tumor cells into the peritoneal cavity (IP) is a good model to study late stages of the disease reproducing the relevant microenvironment of metastatic ovarian cancer [72]. The engraftment rate of IP method varies across different studies. Dobbin et al. and Liu et al. used nude mice for establishment of PDX collections and reported an engraftment rate of 22% and 31%, respectively [76, 80]. Weroha et al. reported a 74% take rate, however, the authors used the more immunodeficient SCID mice [75]. The limitation of the IP model, however, is that mice could become moribund and develop ascites before the PDX tumor reaches the necessary volume to provide enough tissue to perform analyses, as well as to propagate the next generation of PDX models [76]. Subcutaneous implantation, the most common among all heterotopic methods, is technically simpler, faster, and less invasive for animals. Subcutaneous PDX models are easier to monitor compared to orthotopic models. It has been shown that the majority of molecular and genetic characteristics found in original tumors are maintained in subcutaneous models [76, 86], and the tumor engraftment rate is relatively high in this anatomical location (60–80% tumor take rate) [76, 86]. In contrast, Palmer et al. reported only a 20% tumor take rate employing the subcutaneous method; however, the tumor tissue was implanted into nude mice in that study [87]. The main limitation of the subcutaneous model, however, is a lack of relevant tumor microenvironment and the ability to metastasize, thus these models may not accurately mimic the behavior of the human tumor from which they are derived [88]. Consequently, based on the aforementioned reasons, the use of orthotopic PDX models should be prioritized in preclinical studies, especially when evaluating the efficacy of therapies targeting the components of tumor microenvironment.

### Mouse host strains

#### Immunodeficient mouse strains

For successful establishment of PDXs, it is essential to use appropriate immunocompromised murine host strains to avoid rejection of the xenograft. In general, the more immunocompromised the mouse, the higher the engraftment rate [16]. The most frequently used mouse strains includes nude, SCID (severe combined immunodeficient), NOD/SCID (Non-obese diabetic/severe combined immunodeficient), and NSG (NOD/SCID/IL2Rγnul - Non-obese diabetic/severe combined immunodeficient/IL-2 receptor γ-deficient, complete deficiency) mice.

Nude was the first immunodeficient mouse strain used for generation of human xenografts. This murine strain has the least compromised immune system with natural killer (NK) cells present; NK cells often contribute to tumor xenograft rejection. Moreover, nude mice have functional B cells and a tendency to develop a small population of T cells with age [78, 89]. Despite those limitations, nude mice have been successfully used to establish PDXs of ovarian cancer [74, 77, 86].

SCID mice have a better engraftment efficiency compared to nude mice. They lack mature T and B lymphocytes as a result of a spontaneous mutation in the *Prkdc* gene, which disrupts both T and B cell development. The *Prkdc* gene is essential for repair of DNA double-strand breaks induced by radiation [89, 90]. However, remnant NK cells are present in SCID mice [76, 91].

NOD/SCID mice, a cross of SCID mice onto the NOD (nonobese diabetic) genetic background, are the most commonly used strain for PDX generation and have been often used for development of ovarian cancer models [27, 79]. NOD/SCID mice lack mature T and B cells and have impaired NK, macrophage, and dendritic cell function [90]. The NOD/SCID strain has been used to establish more immunosuppressed mice to enhance tumor xenograft acceptance. Such lines include NOG (NOD/SCID/IL2Rγnul - Non-obese diabetic/severe combined immunodeficient/IL-2 receptor γ-deficient, partial deficiency), NOJ (NOD/SCID/Jak3null - Non-obese diabetic/severe combined immunodeficient/Jak3-deficient mice) and NSG. All these mouse strains have a very low tendency to show leakage of T and B cells with age and some tendency to develop lymphoma. A few studies reported development of Epstein-Barr-associated lymphomas following human tissue implantation in these mice [92, 93]. These mouse strains have advantages over other immunocompromised mice in the establishment of PDXs from both solid and hematologic malignancies [89, 90]. Among these highly immunocompromised strains, NSG mice are the most commonly used to establish ovarian cancer PDXs [18, 79, 85, 94].

In 2003 Schultz et al. developed another murine strain called NRG (NOD-Rag1null IL2rγnull) – a further modification of the NOD strain, carrying mutations in the recombination-activating gene *Rag1* and *IL-2* receptor common gamma chain gene [95]. The result of these mutations is an absence of T and B cells, as well as NK cells, respectively [90]. Unlike the SCID strain, NRG mice carry functional *Prkdc* gene and can tolerate higher doses of radiation or chemotherapy, making it a better model for investigating DNA-damaging treatment strategies [96, 97]. So far, NRG mice have been used to establish PDXs from several hematologic malignancies, however these mice are less commonly used to develop PDXs from solid tumors, likely due to a higher popularity of mouse strains on a SCID background [96–98].

Unfortunately, the utilization of highly immunocompromised mice in the generation of PDX models is associated with the development of spontaneous lymphomas [18, 92, 93, 99]. T cells, which play a crucial role in controlling virus infections are absent in immunodeficient mice. Moreover, T cells control oncogenesis associated with Epstein-Barr virus (EBV). Most of the human population is exposed to EBV; however, the infection is under the persistent control of the immune system. Both, T cells and B cells can be infected by EBV and these cells are present in the tumor tissue engrafted into immunocompromised mice. In mice, EBV-positive lymphocytes can expand and develop lymphomas that overgrow tumor xenografts [92, 93, 99]. EBV-associated lymphomas have been described in various types of malignancies [99–102]. To circumvent this problem, it is important to test PDX lines for lymphocytic markers to prevent generation of lymphoproliferative malignancies [23, 92].

#### Humanized mice

One of the limitations of PDX models is that during passaging, tumor-associated stromal cells included in the engrafted human tumor tissue are gradually replaced by the mouse stromal components (extracellular matrix, cancer-associated fibroblasts, macrophages, and leukocytes). This most likely limits complex direct and paracrine interactions between human cancer cells and mouse stromal elements due to insufficient interspecies cross-reactivity, therby affecting tumor growth and its biological behavior [71, 103]. Consequently, the lack of human immune components limits utility of these models to study cancer immunology and to test the efficacy of immunotherapies. To overcome this issue, humanized mouse PDX models has emarged as a promising solution. In this model, patient-derived tumor tissue is implanted into mouse with a human immune system [104].

The humanized mouse models are generated by implantation of human CD34+ hematopoietic stem cells (HSCs) into irradiated NSG mice to enhance the engraftment. HSCs are isolated from peripheral blood, umbilical cord blood or bone marrow. HSCs implantation leads to complete reconstitution of human immune system, since HSCs give rise to all different blood cell lineages. Ideally, to generate humanized PDX model, HSCs should be isolated from the same patient from whom the PDX has been derived. However, there are challenges assocciate with this model, which should be taken into consideration. The growth factors used to mobilize HSCs from bone marrow for isolation from peripheral blood could stimulate tumor progression in a donor patient. Moreover, the yield of HSCs obtained from cancer patients is low, which may require *in vitro* expansion of HSCs [71]. After 10 to 12 weeks posttransplantation, the engraftment can be confirmed by detection of human CD45+ cells in the peripheral blood of NSG mice. More than 25% of human CD45+ cells in peripheral blood is considered as successful engraftment [104].

A different approach to generate humanized mice include the use of peripheral blood mononuclear cells (PBMCs) obtained from donors or tumor infiltrating lymphocytes (TILs) obtained from cancer patients. This method results only in partial reconstitution of human immune system. In addition, within 2–5 weeks post-engraftment, mice develop severe graft-versus-host (GvHD) reaction, which is a significant limitation for long-term studies. Nevertheless, this model could be a sufficient tool in well-designed short-term studies investigating the efficacy immunotherapies [71, 105].

To further improve the generation of humanized mice, several new mouse strains have been developed. For instance, partially humanized N-HSGM3 was generated by crossing HLA-A2/HHD (HHD-II) mice with NSG-SGM3 mice. These mice express stem cell growth factor KITLG, human IL-3 and granulocyte-macrophage colony-stimulating factor (GM-CSF) that allows better engraftment of human HSCs. N-HSGM3 strain displays reduced graft-versus-host disease extending the timeframe of the study [106].

Many available humanized models lack sufficient development and function of human natural killer (NK) and CD8+ T cells. It limits the utilization of these models in studing NK and CD8+ T cell-based immunotherapies. To overcome this issue SRG-15 mice have been developed. These mice express human signal regulatory protein alpha (SIRPA) and human interleukin 15 (IL15). SIRPA enhances the engraftment of HSCs and interleukin 15 is essential for apropriate development and function of circulating and tissue-resident NK and CD8+ T cells [107].

NBSGW (NOD,B6.SCID IL-2rγ-/-KitW41/W41) mice carry mutation in stem cell factor receptor c-Kit supporting HSCs engraftment without prior irradiation of animals. Moreover, NBSGW strain support reconstitution of human immune system folowing transplantation of lower number of HSCs [108, 109].

## PATIENT DERIVED XENOGRAFTS OF OVARIAN CANCER SUBTYPES

### Patient-derived tumor models of high-grade serous ovarian cancer subtype

Several groups have reported establishment of a panel of HGSOC PDX models [27, 73, 79, 80] (Table 1). Comparison of histology and immunohistochemistry of PDX models to the original patient tumors demonstrated conservation of primary tumor morphology, even following multiple serial passages [73, 80]. Moreover, several groups demonstrated that HGSOC PDXs largely represent the molecular landscape of the human HGSOC disease [18, 73, 79, 80].

In his work, Liu et al. compared the CNV profiles of luciferized HGSOC PDXs with the CNV profiles of patient HGSOCs and demonstrated that PDX models matched the CNV profiles of the original tumors with high fidelity. Then the authors compared their CNV data with CNV profiles of publicly available ovarian cancer cell lines, and showed that luciferized PDXs are more clinically relevant models of HGSOC on molecular level than the most established ovarian cancer cell lines [80].

Cybulska et al. reported that the top mutated genes in primary HGSOCs including *TP53*, *BRCA2*, *CSMD3*, *NF1*, *FAT3*, *CDK12*, and *GABRA6* [44], showed similar mutation frequencies in the HGSOC PDXs developed by this group, with the exception of *BRCA1* and *RB1*, which displayed higher frequencies in PDXs vs. patient tumors [79].

In other study, Dong et al. reported that oncogenic pathways commonly dysregulated in HGSOC were maintained without significant changes in PDX models. However, global gene profile analysis between patient tumor and passage 2 of the engrafted HGSOC showed 130 differently expressed genes. These significantly dysregulated genes were associated with a tumor microenvironment, including genes involved in immune modulation, cell-cell adhesion, and stroma reaction. In contrast, the expression of genes associated with oncogenic properties of HGSOC remained unchanged [73]. These findings reflect the observation that changing the tumor microenvironment (from human to mouse) generates selective pressure associated with PDX establishment and adaptation to the mouse host.

Further, selected studies compared the response of HGSOC models vs. their corresponding primary tumors to standard of care chemotherapy (cisplatin/paclitaxel). PDXs derived from patients whose tumors were classified as platinum sensitive displayed significant reduction of tumor size following cisplatin treatment. In contrast, PDXs derived from patients with platinum resistant tumors did not respond to cisplatin treatment [27, 79, 80]. Moreover, HGSOC PDXs treated with multiple cycles of cisplatin became platinum resistant, which mirrored patients’ responses to platinum-based therapy, where the platinum resistance continuously increased following each treatment cycle [27]. In his work, Topp et al. demonstrated that the overexpression of *CCNE1*, *LIN28B*, and *BCL-2* genes correlated with platinum resistance of HGSOC PDXs. These findings are consistent with previous reports implicating these genes in promoting tumor progression and chemotherapy resistance. For instance, *CCNE1* encoding cyclin E1, is amplified in 20% of HGSOCs, which has been associated with chemotherapy resistance and treatment failure in numerous studies [110, 111]. *LIN28B* (miRNA-binding protein) is upregulated in ovarian cancer contributing to maintenance of cancer stem cells, tumor progression and metastasis [112]. *BCL-2* (antiapoptotic protein) was found to be overexpressed in ovarian cancer promoting tumor resistance to cisplatin [113].

As noted previously, approximately 50% of HGSOC display mutation in homologous recombination (HR) genes including *BRCA1* and *BRCA2* [44]. Currently, there is an unmet need for pre-clinical animal models to study therapeutic approaches for the treatment of HR-deficient (HRD) HGSOC. To address this need, George et al. established and characterized a collection of *BRCA*-deficient orthotopic HGSOC PDXs and investigated the ATR-CHK1 checkpoint pathway inhibition as an attractive strategy to activate synthetic lethality in these models [85]. Inhibiting the ATR-CHK1 pathway forces cells to undergo mitosis, which results in mitotic catastrophe and death of cancer cells with damaged DNA (especially those with deficient DNA repair mechanisms) [85, 114]. The study showed that the ATR inhibitor AZD6738, and CHK1 inhibitor MK8776 suppressed PDX tumor growth in monotherapy, however there was no tumor regression observed. The authors concluded that drug combination strategies targeting more than one oncogenic pathway would be a better approach to achieve considerable tumor regression in these models [85].

Bankert et al. established humanized mouse model of ovarian cancer by intraperitoneal injection of cell agregates derived from human tumor that contained CD45+ leukocytes, cytokeratin positive tumor cells, CD3+ T cells and trichrome positive collagen (produced by fibroblasts) into NSG mice [94]. Immunohistochemical analysis of developing tumors in the mouse peritoneal cavity revealed that tumor-associated lymphocytes expressed human-specific lymphocyte markers CD45+, CD3+ T cells, CD20+ B cells, and plasma cell maker CD138+. Further, the authors demonstrated that the established humanized PDX model recapitulates patient’s tumor progression kinetics including ascites development [94].

In different study, Odunsi and colleagues utilized NSG-HHD/SGM3 (N-HSGM3) mice model to test the efficacy of combined immune checkpoint blockers (ICBs) such as anti-PD1/anti-CTLA4 antibodies in combination with adoptively transferred autologous tumor-associated leukocytes/tumor-infiltrating lymphocytes (TALs/TILs) or PBMCs in patient-derived ovarian cancer xenografts. [106]. The presence of TILs in ovarian tumors is associated with improved progression free survival and overall survival [115]. Treatment with autologous TILs may delay tumor growth in ovarian cancer patients. T cells are isolated from patient’s tumor, activated and expanded *in vitro*, and than reinfused to boost endogenous immune responses against tumor [116]. Odunsi et al. demonstrated that treatment with PBMCs and anti-PD-1 antibodies, or TALs and anti-PD-1 antibodies slowed tumor growth rate, however, dual anti-PD1/anti-CTLA4 treatment significantly reduces ovarian cancer growth comparing to control. The authors also reported that the combination treatment with PBMCs was effective despite low frequency of the tumor reactive T cells within PBMC population [106].

Overall, PDX models derived from HGSOC faithfully recapitulate the histologic and molecular features of original patients’ tumors, even following multiple passages. Moreover, these models reflect patient response to chemotherapy with high fidelity. Thus, HGSOC PDXs are valuable pre-clinical models to accelerate progress in new or repurposed drugs validation for clinical use. Additionally, patient derived xenografts established in humanized mice, mirror patient’s primary tumor and could be a useful tool to study cancer immunology and potentially ease optimization of immunotherapies in ovarian cancer.

### Patient-derived tumor models of rare ovarian cancer subtypes

Due to the low frequency of rare tumors in patients and their low tumor take rate, the establishment of pre-clinical models of rare types of ovarian cancer is challenging (Table 1). The lack of well characterized *in vivo* models impairs better understanding of cancer biology and development of new therapies for rare subtypes of ovarian cancer [29, 46, 117]. So far, several research groups generated patient-derived xenografts or organoids of LGSOC, MOC, CCOC, and endometrioid ovarian cancer [29, 118–121]. Similarly as HGSOC PDXs, the PDX models of rare subtypes were histologically and molecularly characterized, and evaluated for treatment response to chemotherapy or more personalized targeted therapies.

Several studies demonstrated that PDX representing endometrioid ovarian cancer are valuable tools to study chemotherapy response and potential mechanisms of chemotherapy resistance. Ricci et al. investigated the mechanism of cisplatin resistance in endometrioid and serous/endometrioid (mixed histotype) PDX models [74, 122]. PDX-bearing mice were initially responsive to cisplatin therapy; however, the following cycles of cisplatin resulted in treatment resistance, mimicking patients’ clinical response [122]. These findings are consistent with studies showing that organoids derived from endometrioid ovarian cancer respond to chemotherapy similarly as their corresponding primary tumors [121]. In their work, Ricci et al. identified differentially expressed genes, which overexpression correlated with resistance to chemotherapy in cisplatin treated vs. control PDXs. The overexpress genes included epithelial-mesenchymal transition (EMT) regulators *(TCF3, CAMK2N1, EGFR*, and *IGFBP4*) and genes promoting stemness (*SMO, DLL1, STAT3,* and *ITGA6*) [122].

Aberrations of the RAS-MAPK signaling pathway play a crucial role in the pathogenesis of LGSOC, which encouraged pre-clinical studies to target components of this pathway [50, 123]. For instance, Fernandez et al. derived 10 LGSOC cell lines from patient tumors and evaluated their *in vitro* sensitivity to four MEK inhibitors including trametinib, selumetinib, binimetinib and refametinib. The authors demonstrated significant differences in sensitivity of LGSOC cell lines to different MEK inhibitors and showed that tramatinib was the most effective agent in inducing apoptosis in LGSOC cells [120]. In other study, tramatinib has been shown to be highly effective in LGSOC subtype with *KRAS* mutations and dysregulated MAPK pathway [29]. De Thaye et al. established a peritoneal metastasis (PM)-PDX model of low-grade serous ovarian cancer, by subperitoneal injection of luciferized patient tumor cells into SCID/Beige mice. The treatment significantly decreased the bioluminescence signal proportional to tumor volume in mice treated with trametinib when compared to the control mice [29]. These studies revealed that established PDXs of rare tumor subtypes such as LGSOC are valuable pre-clinical tools to validate the efficacy of targeted therapies.

Recently, Ricci et al. reported the establishment of two PDX models derived from MOC subtype [117]. Both MOC PDX models were *TP53, BRAF, RAS,* and *PIK3CA* wild type, but displayed amplification of *ERBB2* gene. Ricci et al. evaluated pharmacological profile of established PDXs by testing their response to cytotoxic agents (cisplatin, paclitaxel, trabectedin, oxaliplatin, 5-fluorouracyl) and targeted agents (lapatinib and bevacizumab). The study revealed that cisplatin and paclitaxel treatment inhibited tumor growth in one of the MOC models *in vivo*, which was somewhat inconsistent with patient clinical data showing no response to neoadjuvant platinum-based chemotherapy. One of the potential reasons for this contrasting result was the difference in tumor abundance between patient and mouse model. The patient had an advanced metastatic tumor (stage IV), while mouse had small localized tumor, where the response to treatment is likely to be more favorable. The second MOC PDX was resistant to cisplatin and partially responded to paclitaxel therapy, which better recapitulated the MOC patients’ response to chemotherapy [117]. Further, the authors evaluated the efficacy of trabectedin (Yondelis) using the two MOC PDXs. Ovarian tumors, particularly those with deficient homologues recombination pathway (e.g., *BRCA* deficient cancers) are highly sensitive to trabectedin as shown by numerous studies [124–126]. However, in this study no tumor response to trabectedin was observed likely due to the lack of genomic alterations in the *BRCA* genes [117]. Since MOC subtype displays histological similarity with metastatic mucinous colon cancer [53, 127], Ricci et al. evaluated if MOC PDXs are sensitive to oxaliplatin and 5-fluorouracyl (agents routinely used to treat colon cancer) [128, 129]. However, no treatment response was observed in MOC models following oxaliplatin or 5-fluorouracyl therapy [117]. Within the same study, no anticancer activity was also observed after treatment with the ERBB2 inhibitor lapatinib, despite amplification of *ERBB2* gene in both MOC models [117]. Since MOC is a highly heterogenous tumor with mixed areas of mucinous cystadenoma and borderline lesions [53], it is likely that ERBB2 therapies may be effective only on some tumor cells within MOC leaving the remaining tumor cells intact [130]. Ricci with colleagues have also tested the efficacy of bevacizumab, an antiangiogenic drug approved for maintenance setting in ovarian cancer [131] using the two MOC PDX models *in vivo*; they found this treatment to be moderately active in MOC disease [117]. Overall, this study demonstrated that the pharmacological profile of MOC PDXs partially reflects the treatment response to first line platinum-based chemotherapy. In contrast, the MOC PDXs were largely resistant to other cytotoxic agents used as alternative treatment for ovarian cancer [117]. Expanding the development and pre-clinical testing of anticancer agents with the use of MOC PDX models could better inform treatment choice for MOC patients of this rare cancer subtype.

Availability of CCOC PDX models is limited. Several research groups established only a few CCOC PDXs [74, 75, 77, 86]. Weroha et al. reported successful engraftment and characterization of 168 patient-derived ovarian cancer models encompassing distinct histological subtypes. Within this tumor collection, 10 CCOC PDX models have been established, characterized and available for pre-clinical testing [75]. In other study, Heo et al. reported a successfully engrafted of two CCOC models [77]. Since the abnormal activation of epidermal growth factor receptor (EGFR) constitutes a potential therapeutic target in CCOC subtype [132], Heo et al. tested the efficacy of the EGFR inhibitor erlotinib in CCOC PDXs. As expected, they found that mice bearing CCOC PDX overexpressing EGFR showed a significant decrease in tumor weight following erlotinib treatment, while the PDX model lacking the EGFR expression did not responded to the treatment [77]. In contrast to HGSOC PDXs, there is a limited availability of well characterized PDXs developed from rare EOC subtypes. Nevertheless, these PDXs have emerged as clinically relevant cancer models that offer an opportunity to study the biology of rare ovarian tumors and to test novel targeted therapies.

## PDX APPLICATION IN DRUG REPURPOSING

One of the biggest challenges in anticancer drug discovery is that the vast majority of new agents entering clinical trials do not show acceptable safety and efficacy. The lack of good pre-clinical models to screen new compounds is one of the reasons for these poor results. Many studies suggest that PDXs faithfully recapitulate the diversity of human tumors and are valuable pre-clinical means to predict patients’ drug response [16, 18, 133, 134].

**Figure 3:**
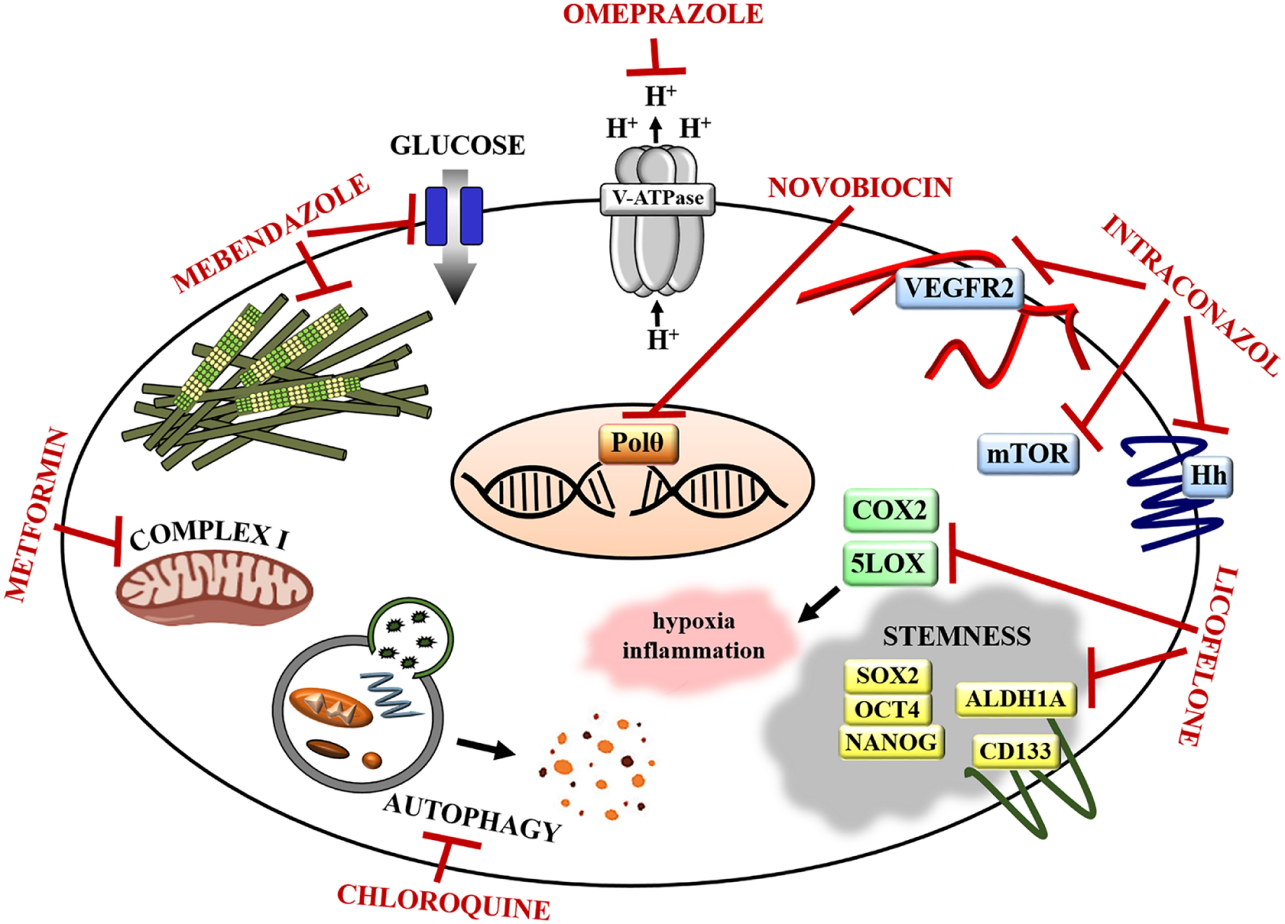
Repurposed drugs targets in ovarian cancer. Omeprazole inhibits V-ATPase, thereby reducing acidification of tumor microenvironment, which sensitizes cancer cells to chemotherapy. Novobiocin inhibits DNA polymerase theta (Polθ) suppressing DNA repair mechanisms and sensitizing cancer cells to PARPi. Itraconazole inhibits Hedgehog (Hh), mTOR, and VEGFR/angiogenesis pathway disrupting tumor vasculature and sensitizing ovarian cancers to paclitaxel. Licofenole attenuates cancer tissue inflammation and hypoxia by inhibiting COX2 and 5LOX. It also reduces the expression of stem cell markers and regulators (OCT4, SOX2, NANOG, ALDH1A, CD133) sensitizing ovarian cancer cells to paclitaxel. Chloroquine disrupts autophagy sensitizing cancer cells to PARPi. Metformin suppresses insulin signals and glucose synthesis via respiratory-chain complex I blockage and sensitizes cancer cells to cisplatin. Mebendazole inhibits microtubule polymerization and glucose uptake by cells effectively killing chemotherapy resistant ovarian tumor cells.

Repurposing (also called repositioning, reprofiling) existing FDA-approved drugs that do not have a primary oncological purpose is a promising alternative strategy to the traditional drug development process. These drugs have known pharmacokinetics and pharmacodynamics and have already been deemed safe in testing in pre-clinical models and humans. Studies show that repurposed drugs are approved within 3–12 years, with costs reduced by 50–60%, in comparison to new compounds [135]. Thus, repurposed drugs help to reduce barriers to clinical trials, and if approved, may be instrumental in improving oncological therapies [11, 135, 136]. Below, we highlight selected repurposed drugs with potential as novel therapies for ovarian cancer (Figure 3). These compounds have been pre-clinically evaluated using PDX models of ovarian cancer [27, 29, 73–75, 77, 79, 80, 85, 86, 117] (Table 1) or other tumor types [137–149] (Table 2).

**Table 2:** Characteristics of repurposed drugs

Repurposed drug	Original drug target	Original indication	Cancer target	Repurposed cancer indication
**Novobiocin**	DNA gyrase	bacterial infections	Polθ	ovarian cancer [28] breast cancer [142]
**Itraconazole**	lanosterol 14a-demethylase biosynthesis of sterols	fungal infections	mTOR Hedgehog VEGFR	ovarian cancer [24] pancreatic cancer [146] glioblastoma [141]
**Mebendazole**	tubulin	parasitic infections	tubulin	ovarian cancer [154] pancreatic cancer [147] breast cancer [149]
**Chloroquine**	heme detoxification	malaria	autophagy	ovarian cancer [26] bladder cancer [140] hepatocellular carcinoma [139]
**Metformine**	gluconeogenesis	type 2 diabetes	complex I	ovarian cancer [162] non-small cell lung cancer [137] colorectal cancer [145]
**Licofelone**	COX-2 5-LOX	osteoarthritis	OCT4 SOX2 NANOG ALDH1A CD133	ovarian cancer [25] melanoma [144] pancreatic cancer [143]
**Omeprazole**	V-ATPase	gastric acidity	V-ATPase	ovarian cancer [168] rectal cancer [148] gastric cancer [138]

Abbreviations: 5-LOX: 5-lipoxygenase; ALDH1A: Aldehyde Dehydrogenase 1 Family Member A1; COX-2: cyclooxygenase-2; Gli1: GLI Family Zinc Finger 1; NANOG: Nanog Homeobox; pERK: phosphorylated extracellular signal-regulated kinase; Polθ: DNA polymerase theta; pS6K1: phosphorylated S6 kinase; SOX2: SRY-Box Transcription Factor 2; VEGFR2: vascular endothelial growth factor receptor 2; V-ATPase: vacuolar H+-ATPase.

### Antibiotic novobiocin (NVB)

Recently Zhou et al. evaluated an anti-cancer activity of the antibiotic novobiocin (NVB) utilizing homologous recombination (HR)-deficient HGSOC PDX models established in NSG mice [28]. NVB is a known inhibitor of DNA gyrase in bacteria and Hsp90 (heat-shock protein 90), as well as topoisomerase II (TOP2), in eukaryotes. Zhou et al. screened over 20,000 bioactive compounds and identified NVB as an inhibitor of DNA polymerase theta (Polθ) in human cells (Figure 3). Polθ plays a crucial role in mutagenic microhomology-mediated end-joining (MMEJ) repair of double strand breaks. This enzyme is frequently overexpressed in HR-deficient breast and ovarian tumors and mediates alternative double strand break repair to compensate the loss of HR. Inhibition of Polθ is synthetically lethal with HR deficiency and leads to cell death in HR-defective tumors. The authors demonstrated that NVB in combination with the PARP inhibitor (PARPi) olaparib was more effective in killing an HR-deficient ovarian cancer cell line than olaparib alone. *In vivo*, NVB in monotherapy led to a significant regression of HR-deficient HGSOC PDX, however when combined with olaparib, NVB caused complete tumor regression. Further, the efficacy of NVB was evaluated in PARPi-resistant PDXs. NVB treatment *in vivo* resulted in tumor growth inhibition, which suggests that there is no cross-resistance between NVB and PARPi. Since Polθ expression is known to correlate with sensitivity to NVB, the authors suggested that the expression of Polθ could be a biomarker for prediction of tumor response to NVB [28]. Based on these pre-clinical data using ovarian PDX models, NVB could be a new promising treatment strategy for ovarian cancer patients with HR-deficient tumors, especially those with innate or acquired PARPi resistance.

### Antifungal agent - itraconazol (ITZ)

Choi et al. showed an anti-tumor potential of itraconazol (ITZ) in two chemoresistant PDX models representing HGSOC subtype and rare, non-epithelial carcinosarcoma of the ovary [24]. Itraconazol is a broad-spectrum inhibitor of lanosterol 14a-demethylase enzyme used for the treatment of candidiasis, aspergillosis, and histoplasmosis [150]. The authors showed that *in vitro* ITZ inhibited proliferation and increased apoptosis of endothelial cell lines (HUVEC and SVEC4-10), but not ovarian cancer cell lines. Moreover, in combination with paclitaxel, an antiproliferative effect of ITZ was enhanced in endothelial cells, though there was no effect on ovarian cancer cells. Additionally, in orthotopic mouse xenografts, established by intraperitoneal implantation of the SKOV3ip1 and HeyA8 cell lines into nude mice, ITZ did not inhibit tumor growth. However, the combination therapy of ITZ and paclitaxel significantly inhibited tumor growth, compared to paclitaxel alone. Choi et al. demonstrated that ITZ monotherapy reduced tumor vasculature (decrease of endothelial cell marker CD31), compared with control, and vasculature reduction was even stronger in tumors treated with a combination of paclitaxel and ITZ. Itraconazol is known to downregulate the mTOR, Hedgehog, and VEGFR2 pathways [151–153] (Figure 3). In agreement with these findings, the authors observed decreased expression of key signaling molecules of those pathways (pS6K1, Gli1, VEGFR2, and pERK) following the treatment with ITZ. *In vivo*, the combination of ITZ with paclitaxel significantly reduced the growth of both ovarian PDXs and decreased the expression of CD31, pS6K1, VEGFR2, and Gli1 [24]. In summary, ITZ demonstrated antiangiogenic and antitumor effect when combined with paclitaxel in PDX models of HGSOC and carcinosarcoma of the ovary, thus the combination of ITZ with chemotherapy, could be a new effective treatment strategy for ovarian cancer patients.

### Antiparasitic agent – mebendazole

HGSOC PDX models have been also utilized to evaluate efficacy of the antiparasitic drug mebendazole (MBZ) [154]. MBZ is a well-tolerated drug used to treat pinworm and other parasitic infections. MBZ binds to tubulin and hinders microtubule polymerization in parasitic cells. It affects cellular structures, glucose absorption, and transport of substances inside the cell, leading to the immobilization and death of parasitic worms [155]. Due to its ability to interact with microtubules, MBZ has been widely studied as a potential anticancer agent (Figure 3). Elayapillai et al. investigated the antitumor effect of MBZ in ovarian cancer cell lines with WT or mutated *TP53*, and observed the inhibition of cell proliferation in all cell lines [154]. For *in vivo* studies, the authors established PDXs from patients with recurrent platinum resistance and different p53 status (*TP53* missense vs. truncating mutations). They reported that MBZ significantly inhibited tumor growth in cisplatin resistant HGSOC PDXs regardless of p53 status, suggesting that MBZ could be an effective therapy for platinum resistant ovarian cancer. The authors reported that the mechanism of MBZ action is associated with microtubule depolymerization and induction of p21 expression leading to p53-independent apoptosis. This study demonstrated that mebendazole shows antitumor efficacy in platinum-resistant HGSOC PDXs, and that highly prevalent *TP53* mutations in ovarian cancer do not have an impact on clinical activity of this antiparasitic agent. Thus, MBZ has a potential to become a new treatment option for ovarian cancer patients [154].

### Antimalarial agent - chloroquine

Santiago-O’Farrill et al. demonstrated an antineoplastic effect of the antimalarial drug chloroquine (CQ) [26]. The authors used HGSOC PDX models, derived from *BRCA*-deficient patient tumors. The activity of CQ is associated with blocking the detoxification process of heme (a product of hemoglobin degradation), which is highly toxic for malaria parasites [156]. In cancer studies, it has been shown that CQ disrupts autophagy (Figure 3). The mechanism by which CQ targets autophagy is not clear, some data indicate that CQ inactivates proteolytic enzymes within lysosomes disrupting the latter steps of autophagy [157]. Santiago-O’Farrill et al. showed that autophagy is a main mechanism of PARPi resistance in ovarian cancer cells, which is in agreement with studies showing that autophagy can protect tumor cells from anticancer treatment [158]. Since the authors observed that the treatment with PARPi olaparib increased autophagy in ovarian cancer cells, they reasoned that concomitant CQ treatment will effectively suppress autophagy reducing survival of cells treated with PARPi [26]. As expected, in this study the treatment combination of olaparib and CQ significantly decreased the viability of 7 ovarian cancer cell lines, and the antitumor effect was stronger in combination therapy vs. single agent therapy. The authors also investigated whether *BRCA* mutation status affects the efficacy of olaparib + CQ combination treatment and concluded that this therapy is effective regardless of *BRCA* status [26]. Further *in vivo* studies with the use of HGSOC PDXs, revealed that CQ as a single agent did not inhibit tumor growth, while treatment with olaparib resulted only in some tumor growth inhibition. In contrast, the combination therapy of CQ and olaparib led to significant tumor growth inhibition [26]. The use of ovarian PDXs in this study was instrumental to show that the antimalarial, antiautophagy agent chloroquine can significantly increase the efficacy of PARP inhibitors. The combination therapy of CQ with PARPi could be taken into the consideration as a novel strategy to overcome PARPi resistance in ovarian cancer patients.

### Antidiabetic agent – metformin

Metformin is used to treat type II diabetes. It blocks glucose synthesis in liver and increases peripheral tissue sensitivity to insulin [159]. An anticancer activity of metformin has been identified by several studies showing that diabetic patients treated with metformin have a lower risk of cancer, including ovarian cancer [160]. The mechanism of antitumor activity of metformin is associated with inhibition of complex I in the mitochondrial respiration chain, leading to defective mitochondrial function and decreased ATP synthesis (Figure 3). Decrease of ATP leads to activation of AMPK (AMP-activated protein kinase) pathway responsible for cellular energy homeostasis. Activated AMPK pathway inhibits mTOR pathway leading to inhibition of cancer cell proliferation and tumor [161]. Studies by Ricci et al. showed that metformin can sensitize ovarian cancer cells to cisplatin [162]. They used three cisplatin sensitive high-grade serous/endometrioid PDXs, expose these to several cycles of cisplatin treatment to generate cisplatin resistant models. Cisplatin resistant PDXs demonstrated different metabolic profiles including upregulated glycolysis, TCA cycle, and urea cycle pathways compared to sensitive models. In addition, oxygen consumption rate and mitochondrial activity increased in cisplatin resistant PDXs. Based on these data, the authors tested if cisplatin resistance could be reversed by a combination of cisplatin and metformin, which inhibits increased mitochondrial metabolism. This study showed that as a single agent metformin lacks anticancer activity, however when combined with cisplatin the antitumor effect is stronger than with cisplatin alone. The mechanism of antitumor efficacy of the combination therapy of metformin and cisplatin has not been further explored. The authors suggested that activation of AMPK by metformin could play a role in sensitizing cancer cells to cisplatin [162]. Overall, these PDX studies illustrated that modulation of tumor metabolism by the antidiabetic agent metformin could be further investigated in chemotherapy resistant ovarian tumors.

### Antiinflammatory agent – licofelone

Hirst et al. studied utilization of the antiinflammatory drug licofelone as a potential treatment for chemotherapy resistant ovarian cancer [25]. Licofelone is a nonsteroidal antiinflammatory drug (NSAID) used to treat osteoarthritis. Licofelone inhibits both cyclooxygenase-2 (COX-2) and 5-lipoxygenase (5-LOX), which metabolize arachidonic acid (AA) and generate prostaglandins (PGs) and hydroxyeicosatetraenoic acids (HETEs), respectively (Figure 3). These compounds mediate many processes, including cell proliferation, differentiation, and inflammation [163]. In ovarian cancer, COX-2 was found to be overexpressed and may be a regulator of tumor progression, whereas 5-LOX promotes hypoxia and inflammation [25, 164, 165]. Gencoglu and colleagues developed a panel of multicellular tumor spheroids (MCTS) by growing epithelial ovarian cancer cells in 3D cell culture. The authors utilized MCTS to screen a drug library to identify drugs with potential antitumor activity and discovered licofelone as a potential anticancer agent [166]. In different study, *in vitro* experiments revealed that licofelone significantly reduced the expression of canonical stem cell markers (*OCT4, SOX2, NANOG*) and cancer stem cell–related genes (*ALDH1A, CD133*) in ovarian cancer cells. In addition, licofenole sensitized these cells to paclitaxel [25]. To perform *in vivo* studies, Hirst et al. established HGSOC PDXs by intraperitoneal implantation of tumor cells isolated from ascites. Paclitaxel treatment *in vivo* increased expression of *ALDH1A* and *CD133*; however, the addition of licofelone to paclitaxel treatment reversed the expression of these genes. In addition, combination therapy of licofelone and paclitaxel significantly improved median survival of PDX-bearing mice, compared to either drug alone. Collectively, the PDX models used by Hirst et al. contributed to the evaluation of the anticancer potential of licofelone, which targets chemoresistant cancer cells with stem cell phenotype. This repurposed drug could be a promising agent to treat ovarian cancer patients with chemotherapy resistant ovarian tumors.

### Proton pump inhibitor (PPI) – omeprazole

Omeprazole is a vacuolar V-ATPase inhibitor that increases intracellular acidification and decreases extracellular acidification, and it is used to treat gastric acidity in gastritis (Figure 3). One characteristic of tumor cells is the alkalization of the cytoplasm and acidification of the tumor microenvironment, which is mediated by V-ATPases that are often overexpressed in various types of carcinoma [167]. It has been shown that acidic tumor microenvironment can affect cytotoxicity of anticancer drugs leading to drug resistance [168, 169]. Lee et al. investigated if V-ATPase inhibitor omeprazole sensitizes tumor cells to chemotherapy by reducing tumor microenvironment acidification [168]. The authors used chemoresistant CCOC PDX model that overexpressed the V-ATPase. *In vitro* studies revealed that the combination therapy of omeprazole and cisplatin or paclitaxel was more cytotoxic than cisplatin or paclitaxel used as a single agents. *In vivo* studies demonstrated that CCOC PDX growth was significantly inhibited after treatment with omeprazole and paclitaxel, relative to paclitaxel alone [168]. This PDX study revealed that omeprazole improves the therapeutic effect of chemotherapy in rare clear cell ovarian cancer subtype. These data also support the rationale to further investigate the anticancer potential of omeprazole in other malignancies.

## CONCLUSIONS

Increasing evidence shows that PDX models derived from various ovarian cancer subtypes capture the diverse nature and dynamic evolution found in primary patient tumors. Moreover, these models faithfully recapitulate the clinical response of patients to standard chemotherapy. The utilization of PDXs representing a common high-grade serous ovarian carcinoma subtype in *de novo* anticancer drug development is consistently increasing. These relevant tumor models could be also successfully used to evaluate the anticancer activity of “old”, already approved drugs without primary oncological purpose. Ovarian PDXs have contributed to the identification of agents that can be repurposed to enhance the therapeutic effect of standard chemotherapy, sensitize cancer cells to chemotherapeutics, or even overcome drug resistance. Unfortunately, the availability of well-characterized PDX models derived from rare ovarian tumor subtypes is still largely limited. This is attributed to the low frequency of rare ovarian tumors and their low engraftment rates in mouse hosts. The establishment of clinically and molecularly annotated PDX models representing distinct ovarian cancer subtypes could considerably aid development of novel therapies or facilitate rapid approval of repurposed drugs for the treatment of gynecological malignancies. Importantly, the establishment of clinically relevant humanized PDX models with a functional immune system, could facilitate the understanding of ovarian cancer immunology and identify novel immunotherapy approaches.
